# CircRNAs in diagnosis, prognosis, and clinicopathological features of multiple myeloma; a systematic review and meta-analysis

**DOI:** 10.1186/s12935-023-03028-z

**Published:** 2023-08-26

**Authors:** Yasin Mirazimi, Amir Hossein Aghayan, Abbasali Keshtkar, Mahsa Mottaghizadeh Jazi, Atefeh Davoudian, Mohammad Rafiee

**Affiliations:** 1 Student Research Committee, Department of Medical Laboratory Sciences, School of Paramedical Sciences, Zanjan University of Medical Sciences, Zanjan, Iran; 2 Department of Health Sciences Education Development School of Public Health, Tehran University of Medical Sciences, Tehran, Iran; 3 Student Research Committee, Faculty of Allied Medicine, Kerman University of Medical Sciences, Kerman, Iran; 4 Deputy of Research and Technology, Zanjan University of Medical sciences, Zanjan, Iran; 5 Department of Medical Laboratory Sciences, School of Paramedical Sciences, Zanjan University of Medical Sciences, Zanjan, Iran

**Keywords:** Circular RNA, Multiple myeloma, Diagnostic, Prognostic, Clinicopathological, Meta-analysis

## Abstract

Unlike improved treatment response in multiple myeloma (MM), the mortality rate in MM is still high. The study’s aim is to investigate the potential role of circRNAs as a new biomarker for diagnosis, prognosis, and clinicopathological features of MM. We identified studies through Web of Science, Scopus, PubMed and ProQuest databases, and Google Scholar to August 2022. The SEN, SPE, PLR, NLR, DOR, and AUC were combined to investigate the diagnostic performance of circRNAs in MM. Also, HR and RR were used for prognostic and clinicopathological indicators, respectively. 12 studies for prognosis, 9 studies about diagnosis, and 13 studies regarding clinicopathological features. The pooled SEN, SPE, DOR, and AUC were 0.82, 0.76, 14.70, and 0.86, respectively for the diagnostic performance of circRNAs. For the prognostic performance, oncogene circRNAs showed a poor prognosis for the patients (HR = 3.71) and tumor suppressor circRNAs indicated a good prognosis (HR = 0.31). Finally, we discovered that dysregulation of circRNAs is associated with poor clinical outcomes in beta-2-microglobulin (RR = 1.56), Durie-Salmon stage (RR = 1.36), and ISS stage (RR = 1.79). Furthermore, the presence of del(17p) and t(4;14) is associated with circRNA dysregulation (RR = 1.44 and 1.44, respectively). Our meta-analysis demonstrates that the expression analysis of circRNAs is valuable for MM’s diagnosis and prognosis determination. Also, dysregulation of circRNAs is associated with poor clinicopathological features and can be used as the applicable biomarkers for evaluating treatment effectiveness.

**Supplementary Information:**

The online version contains supplementary material available at 10.1186/s12935-023-03028-z.

## Introduction

Multiple myeloma (MM) is a type of plasma cell dyscrasia that may start with a monoclonal gammopathy of undetermined significance (MGUS) and progress to plasma cell leukemia and extramedullary myeloma [1]. In MM patients increased secretion of nonfunctional intact immunoglobulins or light chains, can be detected in serum and/or urine [2–4]. Currently, diagnosis, assessment of response to treatment, and minimal residual disease (MRD) in MM patients are made based on the IMWG group criteria [5–7]. Improved treatment response and significantly increased survival have been observed in recent decades, resulting from the use of various therapies in patients with MM [2, 3, 8]. In addition, increased attention must be paid to CRAB (hypercalcemia, renal failure, anemia, and lytic bone lesions) in the multiple myeloma treatment [5]. In recent years, many studies have been done on epigenetic processes involved in the pathogenesis and development of MM, especially studies on diagnostic and prognostic biomarkers with high informative value.

Circular RNAs (circRNAs) are one of the newest types of non-coding RNAs [9]. These single-stranded circular RNAs belong to the long non-coding RNAs, and unlike linear RNAs, they are covalently closed and lack 5’ caps and 3’ tails, which makes them resistant to digestion by RNase and thus more stable [10]. CircRNAs are produced from precursor mRNAs by the back-splicing mechanism [11]. Recent studies in various diseases, especially blood cancers, have shown that circRNAs can play a crucial role as oncogenes or tumor suppressors in intracellular processes by sponging with microRNAs [11–13]. Several studies have investigated the association between circRNAs and pathogenesis, prognosis, diagnosis, and clinicopathological features in MM patients. For example, in 2021, Fan Zhou et al investigated the relationship between 10 circRNAs with high expression and 10 circRNAs with low expression with the clinicopathological features, diagnosis, and prognosis of the disease using microarray analysis and qRT-PCR assays in MM samples [14].

Currently, several methods can be used to diagnose and evaluate the prognosis of MM patients, such as complete blood examination, serum/urine protein detection, bone marrow aspiration/biopsy, flow cytometry, skeletal examination (e.g., X-ray and CT scan), and the ISS and currently revised ISS (R-ISS) systems [5, 15]. Although bone marrow aspiration or biopsy is a well-known approach to confirm the diagnosis, both are quite invasive, expensive, and time-consuming [2]. The using of flow cytometry has promoted the diagnosis of multiple myeloma, but the lack of specific markers and high expensive are limitations of this method [16]. In addition, ISS is a highly accurate method for prognosis determination, but due to the need for systems like interphase fluorescence in situ hybridization and the complex interpretation of the results, these systems are difficult to use [17]. Therefore, it is necessary to develop some minimally invasive and cost-effective methods and discover biomarkers to complement and improve the current strategies for the diagnosis and prognosis of MM.

The purpose of our article is to explore the role of circRNAs in the pathogenesis, development, and response to treatment in patients with MM. A meta-analysis was also carried out using data from included studies to determine the diagnostic and prognostic value of circRNAs for MM. The correlation between circRNAs and clinicopathological features in MM patients was also evaluated.

## Methods

### Eligibility criteria

We accomplished a systematic review, registered on PROSPERO (ID: CRD42022345468). This study was carried out based on PRISMA guidelines [18]. The inclusion criteria were: (A) any sort of peer-reviewed study examining the function of circRNAs (including cellular, circular, and exosomal) in patients with MM, including cohort and case-control studies; (B) studies dealing with aspects of diagnosis, prognosis, progression, and response to treatment of MM. The exclusion criteria were: (A) studies without a complete paper, insufficient data, or just employing an in-silico methodology; (B) non-English-language articles and (C) studies on animals.

#### Information sources

The WOS, Scopus, PubMed, ProQuest databases and Google Scholar were searched for articles published through August 2022. Grey literature sources such as allconferences.com, conferencealerts.com, opengrey, and oatd.org were also searched. The reference lists of included articles were also examined.

### Search strategy

MeSH and non-MeSH keywords used to find related studies were: #1 “RNA, Circular” or “CircRNAs” or “Closed Circular RNA” or “Circular RNA*” ; and #2 “Multiple Myeloma*” or “Myelomas, Multiple” or “Myeloma, Multiple” or “Myeloma, Plasma-Cell” or “Kahler Disease” ; and #3 “Clinicopathologic*” or “clinical-pathological characteristics” ; and #4 “Diagnos*”; and #5 “Sensitivity and Specificity”; and #6 “ROC Curve”; and #7 “Prognos*”; and #8 “hazard ratio”; and #9 “overall survival”; and #10 “Disease-Free Survival”; and #11 “Area Under Curve*”; and #12 “Therapeutic*”; and #13 “Disease Progression*”; and #14 “Risk Stratification”. (The full text of search strategies for all databases is available Additional file 1: S1)

### Selection process

Two researchers (A.A and Y.M) screened the titles and abstracts of all retrieved studies to determine potentially relevant studies for this systematic review. In the next step, the studies’ full text was independently assessed by two researchers to verify the qualified to be included according to the inclusion and exclusion criteria mentioned in Sect. "Eligibility criteria". Any disagreement encountered was resolved by discussion, and if there were unresolvable disagreements, the final decision was made by the third researcher (M.R). Initial screening of the extracted articles was performed using the web-based software Rayyan [19].

### Data collection process

Data extraction of the included articles was performed separately by three researchers (A.A, Y.M, and M.M) based on the data extraction checklist, and if there were unresolvable disagreements, the final decision was made by the fourth researcher (M.R). The WebPlotDigitizer 4.6 software was used to indirectly extract the data from the Kaplan-Meier and receiver operating characteristic (ROC) curves. The methods described by Tierney were used to calculate HR and 95% CI indirectly [20]. However, before the indirect extraction of the data, the authors of the included studies were contacted three times (by email) to obtain information.

### Data items

Three researchers extracted the data by using a pre-specified form. The extracted data included the first author’s name; the name of the circRNA; the year; the number of patients; the number of the control group; changes in circRNA expression; the type of sample; the methods for circRNA analysis (techniques); the control gene; the effect of the circRNA on cell biology; microRNA sponging; and the effect of the circRNA on response to treatment. The required information extracted for the prognosis meta-analysis includes the following: HR with 95% CI for OS (if reported in the article), follow-up time, and survival outcome. Data extracted for the meta-analysis of diagnosis include the following: sensitivity SEN, specificity SPE, cutoff value point, AUC, true positive (TP), false positive (FP), false negative (FN), and true negative (TN). Finally, for the meta-analysis of clinicopathologic features, the data were extracted from the clinicopathologic characteristics tables that are as follows: Gender, B2-MG, albumin, hypercalcemia, renal insufficiency, bone lesions, Durie-Salmon (DS) stage, ISS, and cytogenetic abnormalities such as del (17p), t (4;14), and t (14;16).

### Bias assessment of the studies included

The bias risk assessment was carried out using the Quality Assessment for Studies of Diagnostic Accuracy II (QUADAS II) checklist for diagnostic articles [21], and the Newcastle-Ottawa Scale (NOS) for cohort and case-control articles [22]. The QUADAS II checklist Composed of four key scopes, including patient selection, index test, reference standards, and flow of patients. According to the QUADAS II tool, studies were rated ≥ 6 as high quality and < 6 as low quality (Additional file 2: Fig. S1). The NOS checklist evaluates selection categories, comparability, and outcome (cohort studies) /exposure (case-control studies) categories. articles scoring a 7 as good quality, 5–6 as fair quality, and < 5 as poor quality (Additional file 2: Table. S1). According to the QUADAS II tool, each article receives a maximum of 7 points, and according to the NOS checklist, each article receives a maximum of 9 points.

### Statistical analysis

Extracted data that met the inclusion criteria was synthesized. For diagnostic analysis, the numbers of true positive (TP), false positive (FP), false negative (FN) and true negative (TN) were calculated, and finally the pooled sensitivity, specificity, AUC, PLR, NLR, DOR, 95% CIs, AUC, and heterogeneity were evaluated. The AUC values and their association with diagnostic accuracy are the following: 0.9 to 1.0: excellent, 0.8 to 0.9: very good, 0.7 to 0.8: good, 0.6 to 0.7: sufficient, 0.5 to 0.6: bad and < 0.5: test not useful, and also, good diagnostic tests have positive likelihood ratio (PLR) > 10 and negative likelihood ratio (NLR) < 0.1 [23, 24].

For prognostic analysis, HR and 95% CIs were synthesized to examine the effect of circRNAs on OS. The RR and 95% CIs were used to analyze the clinical value of circRNAs’ association with MM in terms of clinicopathological correlations. Due to methodological heterogeneity in the primary study, the Random Effects Model (REM) was used to combine HR and RR values [25]. The magnitude of association between the study variables and the dysregulated expression of circRNAs and its interpretation areas for the prognostic index (HR) and clinicopathologic characteristics index (RR ) are as follows: 1 to 1.21: trivial (inconsiderable), 1.22 to 1:85: small, 1:86 to 2:99: moderate, 3 or more: large [26]. The chi-square test and the I² statistic were utilized to assess the between-study heterogeneity. If an I² value was < 50%, it was considered to have no significant heterogeneity. To assess the potential source of heterogeneity, subgroup analysis were conducted according to similar features of the included studies, and also, a sensitivity analysis of all the included studies was carried out to find the effect of each article on the final effect of the meta-analysis results. Publication bias was examined quantitatively using the Deek’s funnel plot, Egger’s tests, and Trim and Fills tests. In this study, all meta-analysis was performed with STATA version 14.2 and Meta-Disc software. A p-value < 0.05 was considered statistically significant.

## Results

### Study selection

**Fig. 1:**
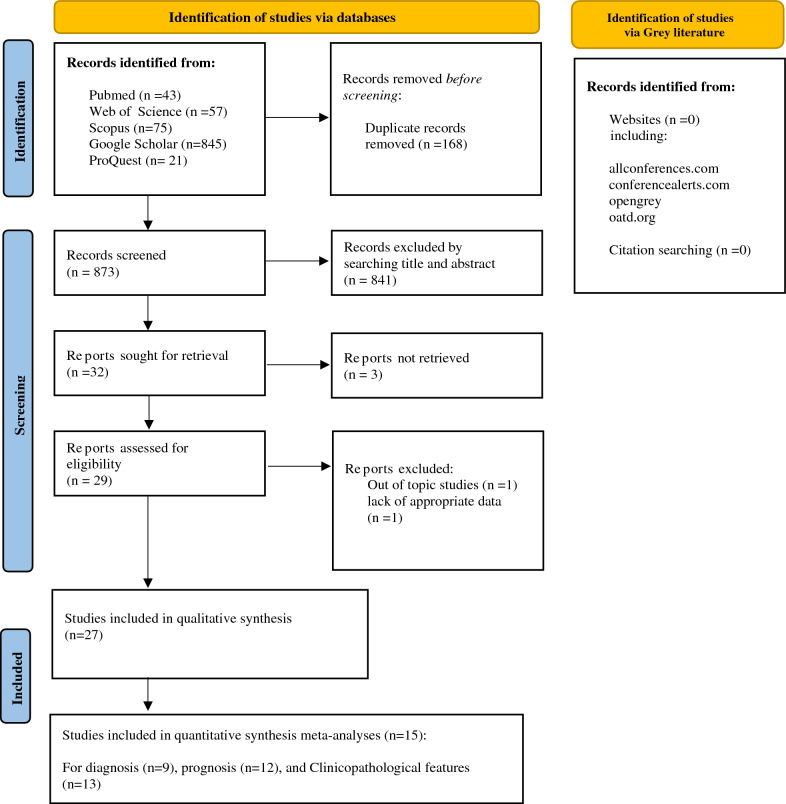
The PRISMA flow diagram for the study selection process

The PRISMA flow diagram [18] of the studies’ selection process is shown in Fig. 1. A total of 1041 studies were extracted via database searches. Prior to the initial screening, 168 articles were removed due to duplication. The title and abstract of 873 articles were initially screened by two researchers, and 841 of them were excluded due to incompatibility with the inclusion and exclusion criteria. 32 studies were selected for full-text examine; 3 full-text studies were not retrieved, and 2 studies were excluded for the reasons described in Fig. 1. Finally, the number of articles included in the qualitative synthesis was 27 [14, 27–52] and the number of articles included in the quantitative synthesis meta-analysis was 15 [14, 27–29, 31–33, 35, 37–41, 48, 50]. Of these, 9 articles were related to the meta-analysis of diagnosis, 12 articles were related to the meta-analysis of prognosis, and 13 articles were related to the meta-analysis of clinicopathological features.

### Study characteristics

All the included articles were published between 2019 and 2022. The total number of patients was 1885, and the study population was exclusively Chinese. Changes in circRNA expression in the studies were measured by the qRT-PCR method. A total of 25 different circRNAs were mentioned; in 10 articles, circRNAs had a tumor-suppressive role, and in 18 articles, circRNAs had an oncogenic role. Table 1 shows the role of circRNAs in cell biology function and their relationship with various microRNAs, as well as the effect of circRNAs in response to treatment. The minimum follow-up period in cohort studies was 14 months, and the maximum was 60 months. In the study of Fan Zhou, 10 circRNAs with high and low expression were measured [14]. To avoid multiplicity [53], one circRNA was selected to perform diagnostic and clinicopathologic features meta-analysis (circ-PTK2) and two circRNAs with oncogenic (circ-PTK2) and tumor suppressive (circ-AFF2) roles to perform prognostic meta-analysis.

**Table 1:** The role of circRNAs in the development of MM and the impact of therapy response

Author’s name	CircRNAs	Gen Symbol	Chr ^a^	Type of circRNA (role)	Impact on functions of cells or biological role	Mechanism	Impact on therapy response
Fang Chen	Circ-0069767	C-KIT protein (CD117) gene	Chr4	Tumor Suppressor	Proliferation/ migration /invasion /apoptosis	Sponging with miR-636 and regulates the expression of K-RAS while the K-RAS gene remained un-mutated	Apoptosis pathway is activated by circ_0069767 and bortezomib is the same. Consequently, the apoptosis rate increases in cells overexpressing circ_0069767
Hongyan Ma	Circ-PSAP	PSAP	Chr10	Oncogene	Cell proliferation/apoptosis	CircPSAP modulates HDAC4 expression by acting as a miR-331-3p sponge, and MiR-331-3p directly targets and inhibits HDAC4	Inhibition of HDAC4 enhances BTZ sensitivity
Yanwei Luo	Circ-MYC (circ-0085533)	MYC	Chr 8	Oncogene	Cell proliferation/ metastasis	–	The expression of circMYC in circulating exosomes is significantly higher in bortezomib-resistant patients than in non-resistant patients. circMYC is significantly associated with response to multiple drugs, such as belinostat (an HDAC inhibitor) and cetuximab
Lin Liu	Circ-0001821	–	Chr8	Oncogene	Proliferation /Apoptosis	Caspase-3 protein expression is lower in patients with higher circ_000182 expression	–
Fangmei Li	Circ-XPO1 (circRNA-102,735)	Exportin 1	Chr2	Oncogene	Proliferation /cell cycle progression /apoptosis	Sponging with MiR-495-3p and miR-495-3p. Regulating the proliferation, apoptosis and cell cycle progression through sponge adsorption of DDIT4	–
Meng Gao	Circ_0007841	Sec61a1	Chr3	Oncogene	Correlates with osteolytic bone destruction in MM	Sponging with miR- 29b-2-5p and miR-199a-3p, overexpression of miR-29b inhibits osteoclast differentiation and reverses osteoclast activation triggered by MM, delaying the progression of MM	miR-29b causes apoptosis of BTZ-induced MM cells by activating the feedback loop of transcription factor Sp1 Consequently, hsa_circ_0007841 may be involved in bortezomib tolerance in MM patients.
Fang Chen	Circ-CDYL	CDYL	–	Oncogene	Cell viability /cell proliferation/ DNA synthesis/ apoptosis	Sponging with miR-1180 and alleviates the repression of miR-1180 on YAP, leading to increased YAP expression	–
Haiyan Liu	Circ-SMARCA5	SMARCA5	Chr 4	Tumor Suppressor	Cell proliferation/ apoptosis	Sponging with miR-767-5p	High expression of circ-SMARCA5 correlates with better response to chemotherapy, which Circ-SMARCA5 might affect the sensitivity of cells to cytotoxic drugs
Hui Zhou	Circ-ITCH	Itchy E3 ubiquitin protein ligase	Chr 20	Tumor Suppressor	Cell proliferation/ apoptosis	–	–
Shans an Yu	Circ‑MYBL2	MYBL2	–	Tumor Suppressor	Cell viability/DNA synthesis /cell cycle progression	Circ-MYBL2 facilitates binding of cyclin F to MYBL2, attenuates phosphorylation and activation of MYBL2, and thereby inhibits transcription of a number of known proliferation-related oncogenes	–
Xiao Liu	Circ-101,237	CDK8	Chr13	Oncogene	–	11 differentially expressed miRNA and 10 candidate mRNAs interact with hsa_circRNA_101237	hsa_circRNA_101237 upregulation may be one mechanism of bortezomib resistance in MM patients
Xingxig Gonga	Circ-0087776	-		Tumor Suppressor	–	–	The expression level of hsa_circ_0087776 is significantly higher after chemotherapy than before chemotherapy
Yan Li	Circ-KCNQ5 (circ-0007165)	Potassium channel gene (KCNQ5)	Chr6	Oncogene	Proliferation/migration/ invasion/ glycolysis/ apoptosis	Sponging with miR-335-5p and MiR-335-5p, interacts with circKCNQ5 and is also able to affect BRD4 in MM cells	–
Manya Yu	Circ-ATP10A	ATP10A	Chr15	Oncogene	Angiogenesis/ marrow microvessel density (MVD)	Sponging with miR-6758-3p/ miR 3977/miR- 6804-3p/ miR-1266-3p/miR-3620-3p, to regulate the expression of VEGFB, HIF1A, PDGFA, and the FGF family	-
Runjie Sun	Circ-G042080	-	Chr2	Oncogene	Autophagy/ proliferation	Sponging with miR-4268/ TLR4 axis, and circ-G042080 activates TLR4 and thereby induces autophagic death in cardiomyocytes in M.M patients	-
Wei Fang	circRNA arginine-glutamic acid dipeptide repeats (Circ-RERE or circ-0009581)	RERE	Chr1	Oncogene	Proliferation /BTZ resistance	circRERE with sponging miR-152-3p, to generate expression regulation of CD47, MiR-152-3p promotes susceptibility of MM cells to BTZ by targeting CD47	The upregulation of circRERE in BTZ-resistant MM samples and cells, circRERE facilitates the resistance of MM to BTZ by sponging miR-152-3p and upregulating CD47
Xiaoya Li	circ_0058063	–	–	Oncogene	Proliferation /migration/ invasion/ apoptosis	Sponging with miR- 635	–
Yan Wang	Circ-0007841	–	–	Oncogene	Proliferation/cell cycle/metastasis /apoptosis	circ_0007841 accelerates progression of MM by targeting miR-338-3p, and BRD4 directly bind to miR-338-3p (promotes activation of PI3K/AKT signaling via miR-338-3p/BRD4 axis), also reports that miR-338-3p suppresses proliferation and accelerates apoptosis of MM cells via CDK4	–
Yashu Feng	Circ-0000190	–	chr1	Tumor Suppressor	Cell viability/ proliferation / apoptosis	Sponging with miR-767-5p, and Mitogen-activated protein kinase 4 (MAPK4) is a direct target of miR-767-5p	–
Yongsheng Xiang	Circ-0000190	–	–	Tumor Suppressor	–	Sponging with miR-767-5p	No difference in circ_0000190 expression between CR patients and non-CR patients
Yu-Hui Zhu	Circ-0000190	–	–	Tumor Suppressor	Cell cycle /apoptosis/ migration /invasion	Sponging with MiR-301a, and the activation of JAK1/STAT3 pathway promotes by miR-301a	Icaritin treatment blocks malignant development of MM by increasing the expression of circ_0000190
Fang Liu	Circ-CCT3 (Circ-0000142)	–	Chr1	Oncogene	Proliferation/migration/ invasion/ apoptosis	Enhances the proliferation and metastasis of MM cells by modulating the miR-610/AKT3 axis	–
Fan Zhou	10 upregulated 10 downregulated	–	Chromosomes 8,2,61,3,16,10,13, x,20,4,15,11	Oncogene And Tumor Suppressor	–	circ-AFF2 might sponge miR-638 and inhibit the oncogenic function of miR-638 in MM. circ-PTK2 might act as sponge for anti-oncogenic miR-1298-5p and promotes the neoplastic progression in MM	circ-PTK2 and circ-RNF217 correlate with poor treatment response and survival, whereas circ-AFF2 predicts good treatment response and survival in MM patients
Xiaozhu Tang	Circ-BUB1B	BUB1B	Chr15	Oncogene	Proliferation /drug resistance	The circular form of the BUB1B gene encodes a novel 544-amino acid protein in MM cells called circBUB1B_544aa. circBUB1B_544aa and BUB1B play a synergistic role in triggering CIN in MM by activating CEP170, leading to MM cell proliferation and drug resistance	circBUB1B in tissue from patients with relapse (RP) is significantly higher than in comparable tissue from newly diagnosed patients (ND) and normal tissue (NP)
Xiaozhu Tang	Circ-HNRNPU (circ-0017272)	HNRNPU	Chr1	Oncogene	Proliferation/ cell cycle	MM Cells secrete circHNRNPU, which encodes a protein called circHNRNPU_603aa. Overexpression of circHNRNPU_603aa promotes MM cell proliferation and circHNRNPU_603aa competitively inhibits c-Myc ubiquitin, and so stabilize c-Myc in MM Increase in circHNRNPU is associated with poor outcome in MM patients	–
Jianhua Liu	Circ-ITCH	Itchy E3 ubiquitin protein ligase	–	Tumor Suppressor	Proliferation /apoptosis /BTZ resistance	Sponging with miR-615-3p/PRKCD axis	CircITCH overexpression enhances the sensitivity of MM cells to BTZ through miR-615-3p/PRKCD axis
Lianguo Xue	Circ-0058058	ATIC ^b^	Chr2	Oncogene	Proliferation/ angiogenesis /metastasis/ apoptosis	Sponging with miR‑338‑3p/ATG14 axis	–

^a^Chromosome; ^b^ 5-Aminoimidazole-4-Carboxamide Ribonucleotide Formyltransferase/IMP Cyclohydrolase

### Results of syntheses

#### The prognostic performance of circRNAs in multiple myeloma

**Fig. 2:**
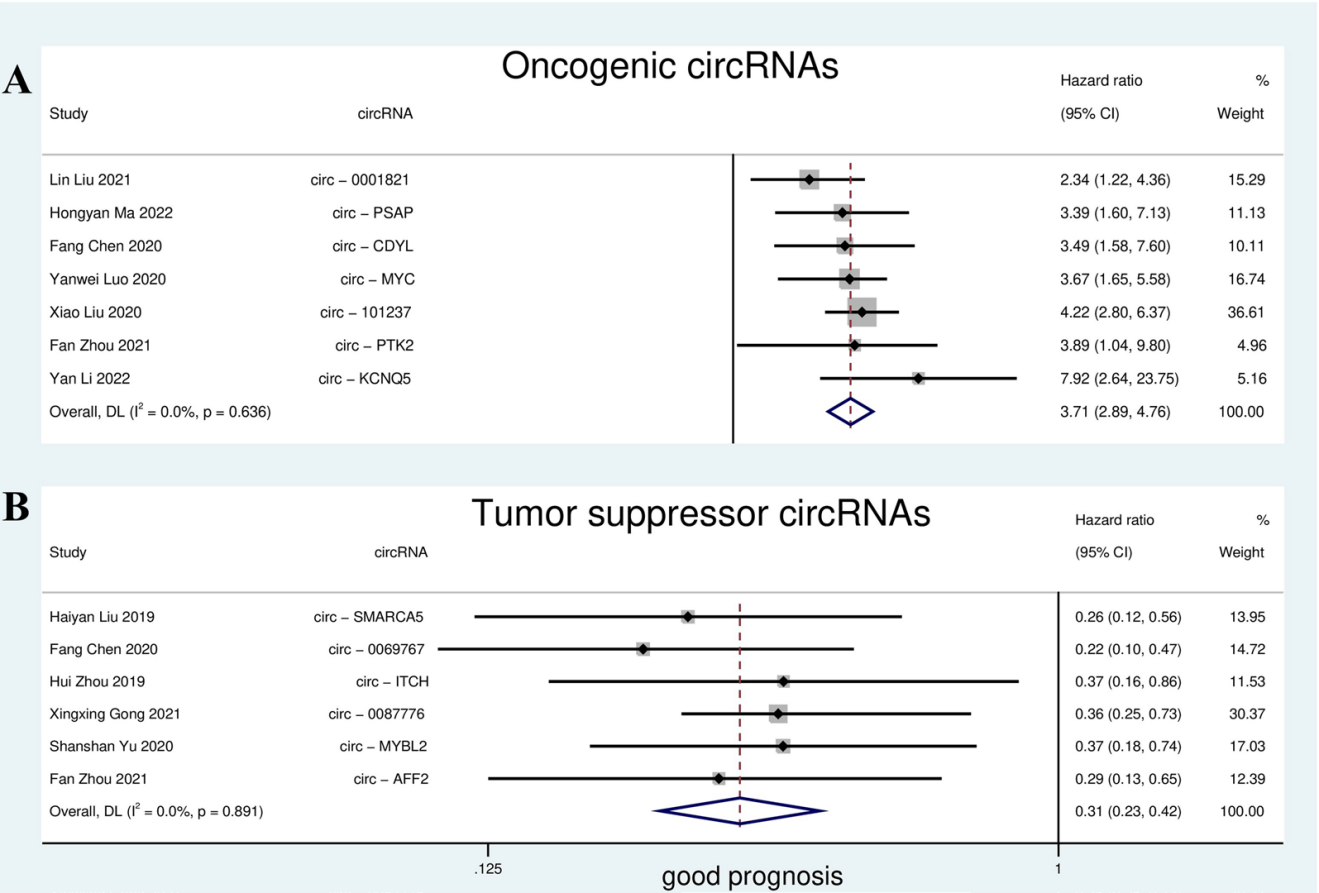
Forest plots for the prognostic value of circRNAs in overall survival (OS) of MM patients. Oncogenic circRNAs (High-expressing) indicate worse prognosis (**A**) and tumor suppressor circRNAs (Low-expressing) indicate good prognosis in the MM patients (**B**)

After reading the details of the I^2^ included articles, the prognostic value of circRNAs was assessed. The main characteristics of prognostic studies are shown in Table 2. CircRNAs with an oncogenic role in MM patients were found in 7 studies and were negatively associated with the patients’ prognosis. After meta-analysis, oncogene circRNAs showed poor prognosis for MM patients (high expression group vs. low expression group: HR = 3.71; 95% CI 2.89 to 4.76); also, I^2^ = 0 showed that the results have low heterogeneity (Fig. 2A). Meanwhile, another 6 studies reported that circRNAs are tumor suppressors in MM patients and have a positive association with patient prognosis. Tumor suppressor circRNAs indicated a good prognosis for MM patients (high expression group vs. low expression group: HR = 0.31; 95% CI 0.23 to 0.42) and I^2^ = 0 indicated that the results have low heterogeneity (Fig. 2B).

**Table 2:** Main characteristics of the prognostic studies

Author’s name	Year	CircRNAs (n = 13)	MM^a^ patiens size	Sample type	Method	Survival indicator (OS^b^)		HR Extraction	Follow up^*^	NOS^e^
						HR^c^ (95% CI^d^)	P value			
Fang Chen	2020	Circ-0069767	66	Bone marrow	qRT-PCR	0.22 (0.1–0.47)	0.0001	Indirectly	60	7
Hongyan Ma	2022	Circ-PSAP	50	Bone marrow	qRT-PCR	3.39 (0.99–3.88)	/	Indirectly	60	7
Haiyan Liu	2019	Circ-SMARCA5	105	Bone marrow	qRT-PCR	0.259 (0.119–0.565)	0.001	Directly	40	8
Lin Liu	2021	Circ-0001821	115	Bone marrow	qRT-PCR	2.342 (1.217–4.355)	0.031	Directly	60	8
Fang Chen	2020	Circ-CDYL	72	Bone marrow and PB^f^	qRT-PCR	3.49 (1.59–7.60)	0.0017	Indirectly	60	7
Hui Zhou	2019	Circ-ITCH	92	Bone marrow	qRT-PCR	0.367 (0.156–0.865)	0.018	Directly	36	8
Xingxing Gong	2021	Circ- 0087776	136	PB (serum)	qRT-PCR	4.228 (2.564–6.974)	0.001	Directly	–	7
Shanshan Yu	2020	Circ‑ MYBL2	89	Bone marrow and serum	qRT-PCR	0.37 (0.18–0.74)	0.0052	Indirectly	50	6
Yanwei Luo	2020	Circ-MYC	122	PB (serum)	qRT-PCR	3.67 (1.65–5.58)	0.0001	Directly	60	8
Xiao Liu	2020	Circ-101,237	143	Bone marrow	qRT-PCR	4.22 (1.05–3.71)	0.035	Indirectly	60	7
Fan Zhou	2021	Circ-PTK2	60	Bone marrow	qRT-PCR	3.89 (1.54–9.79)	0.004	Indirectly	40	6
Fan Zhou	2021	Circ-AFF2	60	Bone marrow	qRT-PCR	0.29 (0.15–0.65)	0.003	Indirectly	40	6
Yan Li	2022	Circ-KCNQ5	43	Bone marrow	qRT-PCR	7.96 (2.65–23.80)	0.0001	Indirectly	60	8

^a^ Multiple myeloma; ^b^ Overall survival; ^c^ Hazard ratio; ^d^ 95% confidence interval; ^e^ Newcastle-Ottawa Scale; ^f^ Peripheral blood

^*^ Months

#### The diagnostic performance of circRNAs in multiple myeloma

**Fig. 3:**
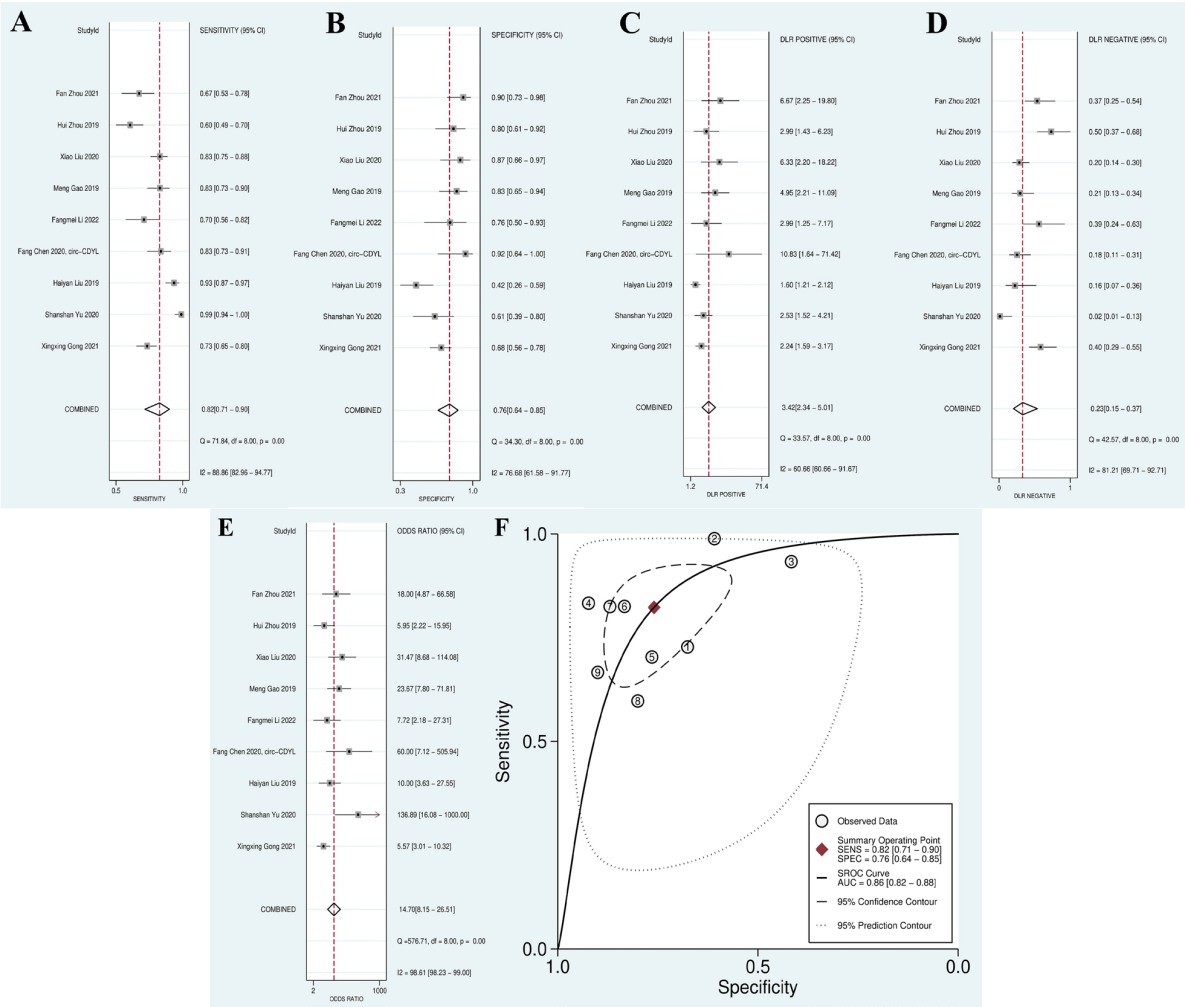
Forest plots of the combined Sensitivity (SEN) (**A**), Specificity (SPE) (**B**), Positive likelihood ratio (PLR) (**C**), Negative likelihood ratio (NLR) (**D**), odds ratio (DOR) (**E**) and the SROC curve (**F**) in diagnostic value analysis

The SEN and SPE of circRNAs for the diagnosis of MM are shown in Table 3. The pooled sensitivity and specificity were 0.82 (95% CI 0.71–0.90) and 0.76 (95% CI 0.64–0.85), respectively (Fig. 3A, B). In addition, the pooled PLR, NLR, and DOR were 3.42 (95% CI 2.34–5.01), 0.23 (95% CI: 0.15–0.37), and 14.70 (95% CI 8.15–26.51), respectively (Fig. 3C, D and E). Also, the area under the summary ROC (SROC) curve of circRNAs for distinguishing MM from healthy controls was 0.86 (95% CI 0.82–0.88) (Fig. 3F). Furthermore, the Fagan’s nomogram (to describe the post-test probabilities of disease in MM patients) (Additional file 3: Fig S1), the likelihood ratio scattergram (Additional file 3: Fig. S1/Fig. 1A), and the Probability Modifying Plot (Additional file 3: Fig. S2/Fig. 1B) have been used in the clinical application of circRNAs.

**Table 3:** Main characteristics of the diagnostic studies

Study name	Year	CircRNAs (n = 9)	MM^a^ patients size	Control size	Sample type	Control gene	Cut- off value	Diagnostic indexes			QUADAS Score
								AUC^b^	Sen^c^	Spe^d^	
Xingxing Gong	2021	Circ-0087776	136	74	PB^e^ (Serum)	18 S	–	0.72	72.8	67.6	4
Shanshan Yu	2020	Circ‑MYBL2	89	23	Bone marrow and serum	GAPDH	–	0.83	98.9	62.7	6
Haiyan Liu	2019	Circ-SMARCA5	105	36	Bone marrow	GAPDH	2.242	0.71	93.3	41.7	7
Fang Chen	2020	Circ-CDYL	72	13	Bone marrow and PB	–	–	0.89	83.4	92.2	4
Fangmei Li	2022	Circ-XPO1 (Circ-102,735)	54	17	Bone marrow	β‑actin	–	0.78	71.2	76.8	5
Meng Gao	2019	Circ-0007841	86	30	Bone marrow	GAPDH	–	0.90	82.6	83.4	6
Xiao Liu	2020	Circ-101,237	143	23	Bone marrow	β-actin	–	0.92	82.8	86.4	3
Hui Zhou	2019	Circ-ITCH	92	30	Bone marrow	GAPDH	–	0.80	59.8	80	7
Fan Zhou	2021	Circ-PTK2	60	30	Bone marrow	GAPDH	–	0.81	66.8	90	7

^a^ Multiple myeloma; ^b^ The area under the receiver operating characteristic curve; ^c^ Sensitivity; ^d^ Specificity; ^e^ Peripheral blood

#### Subgroup analysis

Due to significant heterogeneity, subgroup analyses were carried out according to the function of circRNAs (oncogenic or tumor suppressor) and quality studies based on QUADAS II (high or low) to evaluate the potential sources of heterogeneity. As shown in Table 4, oncogene circRNAs achieve a higher diagnostic performance than tumor suppressor circRNAs, with AUC values of 0.88 and 0.77, respectively. Moreover, a comparison of quality studies shows that the AUC (0.86 vs. 0.81) and the DOR (15.64 vs. 13.28) of high-quality studies were higher than those of low-quality studies (Forest plots of subgroup analysis are in the Additional file 3: Fig. S2).

**Table 4:** Subgroup analysis for diagnostic meta-analysis ^a^ Positive likelihood ratio; ^b^ Negative likelihood ratio; ^c^ Diagnostic odds ratio; ^d^ The area under the receiver operating characteristic curve

Subgroups	No. of studies	Sensitivity (95% CI^e^)	Specificity (95% CI)	PLR ^a^ (95% CI)	NLR^b^ (95% CI)	DOR^c^ (95% CI)	I^2^	AUC^d^
Total study included	9	0.82 (0.71–0.90) 0.78 (0.69–0.85)	0.76 (0.64–0.85) 0.78 (0.66–0.86)	3.42 (2.34–5.01) 3.50 (2.28–5.39)	0.23 (0.15–0.37) 0.28 (0.21–0.39)	14.70 (8.15–26.51) 12.43 (7.15–21.61)	98.61% 92.89%	0.86 0.85
Outliers excluded	8							
Function of circRNA:		0.79 (0.74–0.83) 0.81 (0.76–0.84)	0.86 (0.78–0.92) 0.63 (0.55–0.71)	4.98 (3.17–7.84) 2.10 (1.58–2.78)	0.26 (0.19–0.36) 0.26 (0.13–0.52)	19.96 (11.03–36.10) 9.68 (4.05–23.12)	0.00% 64.60%	0.88 0.77
Oncogene	5							
Tumor suppressor	4							
Quality of studies:		0.81 (0.77–0.85) 0.79 (0.73–0.82)	0.70 (0.62–0.78) 0.75 (0.66–0.82)	2.99 (1.69–5.29) 3.56 (1.81–7.01)	0.24 (0.13–0.45) 0.28 (0.18–0.43)	15.64 (7.00–34.97) 13.28 (4.66–37.82)	53.80% 67.10%	0.86 0.81
High	5							
Low	4							

#### The clinicopathological significance of circRNAs in multiple myeloma

Regarding the clinicopathological characteristics, 13 studies were included in our meta-analysis. We looked at the relationship between circRNA expression and clinicopathological features like gender, B2-MG, albumin, hypercalcemia, renal insufficiency, bone lesions, DS stages, ISS stages, and cytogenetic abnormalities like del(17p), t(4;14), and t(14;16) (at least five studies were looked at for each feature) (Table 5). Dysregulation of circRNAs has been associated with adverse clinical features DS stage; RR = 1.36, 95%CI 1.13–1.64, ISS stage; RR = 1.79, 95%CI 1.46–2.18, B2-M; RR = 1.56, 95%CI 1.20–2.03, (Additional file 4: Fig S1). Notably, there was no association between circRNA expression and other clinicopathological features such as gender, albumin, hypercalcemia, renal insufficiency, bone lesions, and t(14;16) (Forest plots of other clinicopathological features are in the Additional file 4: Fig. S2). Furthermore, our results indicate that the presence of del(17p) and t(4;14) is associated with dysregulation of circRNAs with RR = 1.44, 95% CI 1.18–1.75, and RR = 1.44, 95% CI 1.24–1.68, respectively (Additional file 4: Fig. S1).

**Table 5:** Correlation between circRNAs and clinicopathological features of MM

clinicopathological parameters	No. of studies	p value	Risk ratio (95%CI)	I^2^(%)
Gender (male/female)	12	0.977	1.00 (0.89 to 1.12)	0
B2-MG ^a^ (abnormal/normal)	5	**0.001**	1.56 (1.20 to 2.03)	51.2
Albumin (abnormal/normal)	5	0.259	1.12 (0.92 to 1.35)	0
Hypercalcemia (yes/no)	7	0.510	0.94 (0.80 to 1.12)	11.4
Renal insufficiency (yes/no)	5	0.774	1.04 (0.80 to 1.35)	52
Bone lesions (yes/no)	9	0.464	1.06 (0.90 to 1.26)	32.8
DS stage ^b^ (III/I,II)	10	**0.001**	1.36 (1.13 to 1.64)	38.1
ISS stage ^c^ (III/I,II)	10	**0.000**	1.79 (1.46 to 2.18)	47.3
Del(17p) ^d^ (yes/no)	6	**0.000**	1.44 (1.18 to 1.75)	26
t (4–14) (yes/ no)	6	**0.000**	1.44 (1.24 to 1.68)	0
t (14–16) (yes/ no)	5	0.957	1.01 (0.77 to 1.31)	21.2

P values less than 0.05 are shown in bold

^a^ Beta 2 Microglobulin; ^b^ Durie-Salmon stage; ^c^ International Staging System; ^d^ Deletion

## Sensitivity analysis and publication bias evaluation

### Related to prognosis

Low publication bias was found in the combined prognostic effects of two groups of oncogenes and tumor suppressors, as shown in Additional file 5: Fig S1/Fig. 1A C (Egger’s test, P values of 0.752 and 0.505, respectively). The Trim and Fill method was used to better estimate the potential effects of publication bias, and like Egger’s test, publication bias was not significant (Additional file 5: Fig. S1/Fig. 1B, D).

The sensitivity analysis was performed in the 2 subgroups of oncogene and tumor suppressor, and there was no outlier study (Additional file 5: Fig. S2/Fig. 2A, B), indicating our results were not significantly to be affected by any individual of the included studies.

### Related to diagnosis

The sensitivity analysis showed that one included study (Shanshan Yu, 2020) had a big impact on the pooled results (Additional file 5: Fig. Fig. S3/Fig. 3A). After removing this study, the I^2^ value for the heterogeneity of DOR decreased from 98.61 to 92.89% (Table 4). Nonetheless, the pooled diagnostic values were comparable with those of the total studies (AUC: 0.86 vs. 0.85), showing that our results were relatively robust and not significantly to be affected by any individual of the included studies.

As displayed in Additional file 5: Fig. 3/Fig. 3B, non-considerable publication bias was detected in the combined diagnostic effects (Deek’s funnel plot, p value: 0.08).

## Discussion

CircRNAs play a role in a wide range of cell biology by sponging with various microRNAs in MM cells [51]. As shown in Table 1, increasing or decreasing expression of circRNAs in MM cells ultimately affects the processes of proliferation, apoptosis, metastasis, cell cycle regulation, and response to treatment. Interestingly, in contrast to other studies, the study by Fang Chen [27] showed that circ-0069767, as a tumor suppressor, has increased expression in MM cells. The increased expression of this circRNA leads to a decrease in proliferation, migration, and invasion and an increase in apoptosis in MM cells. On the other hand, interestingly, some circRNAs have the ability to translate and produce proteins [54, 55]. CircRNAs through different mechanisms can be translated and produce proteins such as N6 methyladenosine modification or via the internal ribosome entry site (IRES), regions that elevate direct binding of initial factors to circular RNAs [56–59]. Two studies by Xiaozhu Tang et al. have shown that circBUB1B and circ-HNRNPU have the ability to translate and produce circBUB1B_544aa and circHNRNPU_603aa proteins, respectively [43, 44].

Several primary studies have demonstrated the prognostic value of circRNAs in MM. This prognostic meta-analysis included 12 studies and 1093 MM patients. MM patients with increased expression of oncogenic circRNAs had a poorer OS and a nearly 4-fold higher risk of death than the control group (HR = 3.71); moreover, increased expression of tumor suppressor circRNAs are associated with a favorable OS, and almost 70% of the risk of death in this group is lower than the control group (HR = 0.31). So finally, According to the mentioned interpretation areas [26], a large correlation was observed between increased expression of oncogenic circRNAs and OS and a large correlation between increased expression of tumor suppressor circRNAs and OS. All these results indicate that circular RNAs play a role as novel biomarkers in predicting OS in patients with multiple myeloma.

Our results showed that circRNAs are diagnostic promising biomarkers for MM, with a combined AUC: 0.86 and DOR: 14.70, that larger AUC represents greater diagnostic value of each variable [23], and a higher DOR, as an important index used in meta-analysis of diagnostic studies, represents a more valuable indicator with better diagnostic efficacy (Fig. 3E). Moreover, the pooled sensitivity and specificity of circRNAs were 0.82 and 0.76, respectively, implying that circular RNAs represents good diagnostic accuracy. In addition, PLR values were 3.42, which means circRNA expression changes (positive results) happen 3.42 times more in a multiple myeloma patient than a patient without the multiple myeloma, and NLR values were 0.23, which means the probability of a negative test in a non-patient is 4.34 times greater than that of a negative test in an M.M patient. As circRNAs with diverse expression statuses may exert different functions in MM, we’ve performed subgroup analyses. Stratified analysis based on the function of circRNA showed better diagnostic accuracy for oncogene circRNAs than tumor suppressor circRNAs for MM. Moreover, based on quality subgrouping, it revealed that high-quality studies achieved a higher diagnostic performance than low-quality studies.

Heterogeneity is unavoidable in a meta-analysis and was therefore also evident in our meta-analysis. We also explored the potential factors responsible for heterogeneity using the sensitivity analysis and the subgroup analysis. The sensitivity analysis indicated that one study was an outlier, but further investigation revealed that the heterogeneity of our data was acceptable, and the combined effects were reliable. The subgroup analysis traced the different factors, such as circRNAs expression level, and showed that the function of circRNAs may be a major cause of heterogeneity. Aiding with clinical decision-making is one of the important key features of a novel biomarker. Therefore, likelihood ratios (negative and positive) and post-test probabilities are two useful parameters for medical professionals, because they provide information about the likelihood that a patient with a positive or negative test actually has MM or not. This study demonstrated the clinical applicability of two positive and negative likelihood ratio indices in the diagnosis of MM. PLR > 10 and negative likelihood ratio NLR < 0.1 indicate good diagnostic accuracy of test [23, 24]. In addition, the Fagan nomogram was used to describe the post‐test probabilities of disease in the MM patients. If the prior probability of MM is 20%, the post-test probability of MM would reach 46% if the circRNA test is positive, and if the circRNA test is negative, this would mean that the post-test probability of MM would drop to 6%.

For the final interpretation of the clinicopathological features, the RR was chosen for the report because, if the odds ratio were reported, the association between circRNAs and clinicopathological features would be exaggerated [60]. Our results show a small but significant association between aberrant expression of circRNAs and elevated ISS and DS stages and B2-MG, which indirectly reflect the status of MM patients. Furthermore, the presence of del(17p) and t(4;14) has a small but significant association with abnormal circRNA expression.

## Conclusion

According to the importance of MM diagnosis and the determination of the prognosis for effective management, our review suggests measuring the changes in the expression of circRNAs as a specific and valuable marker related to the prognosis and diagnosis of MM. Also, the changes in the expression of circRNAs can be associated with poor clinicopathological features and can be used as valuable markers for investigation of treatment effectiveness and clinical diagnosis. Through future studies, circRNAs can be considered important targets for the efficient treatment of MM.

## Limitations of the review

However, our current meta-analysis still had the following limitations: First, the studies were all from China, which may circumscribe the generalization of these findings and lead to population selection bias. The second is the lack of access to the cutoff to check the threshold effect. Third, heterogeneity is still a vital issue in this meta-analysis, although we carried out subgroup analysis to explore possible sources. Fourth, the number of included studies is relatively small, which may give the appearance of bias. Fifth, due to the small number of studies, individual analysis of more subgroups was limited. The sixth reason is that articles with positive results are more likely to be published, which may increase overall diagnostic accuracy and the seventh, Due to the linguistic restrictions we only accepted articles in English (at least in the abstract), which may have influenced our results.

### Supplementary information

**Additional file 1**: The full text of search strategies for all databases.

**Additional file 2**: **Figure S1.** Quality assessment by the QUADAS II. Each bias risk item for each included study (A), each bias risk item is presented as a percentage for all included studies (B). **Table S1.** Supplemental Content, which illustrates study quality assessed via the Newcastle-Ottawa Scale checklist.

**Additional file 3**: **Figure S1.** Likelihood ratio scattergram (A), Relationship between pre and post-test probability based on the likelihood of a positive (above digonal line) or negative (below diagonal line) test (B), Fagan’s nomogram to describe the effect of circRNAs on the diagnosis of MM (C). **Figure S2.** Forest plots of Subgroup analysis based on DOR. Subgroup analysis based on type of circRNAs (A), Subgroup analysis based on quadas score (B).

**Additional file 4**: **Figure S1.** Forest plots of DS stage (A), ISS stage (B), B2-MG (C), del(17p) (D) and t(4;14) (E) in the clinicopathological features association analysis with circRNAs in MM patients. **Figure S2** Forest plots of other clinicopathological parameters.

**Additional file 5**: **Figure S1.** Publication bias evaluation for prognostic studies. Egger’s test (A) and Trim and fill (B) method for oncogene circRNAs. Egger’s test (C) and Trim and fill (D) method for tumor suppressor circRNAs. **Figure S2.** Sensitivity analysis for oncogene (A) and tumor suppressor (B) circRNAs.**Figure S3.** Sensitivity analysis (A) and Deeks’ funnel plot (B) for diagnostic studies.

## Data Availability

Not applicable.

## Abbreviations

MM: Multiple myeloma
OS: Overall survival
SEN: Sensitivity
SPE: Specificity
PLR: Positive likelihood ratio
NLR: Negative likelihood ratio
DOR: Diagnostic odds ratio
AUC: Area under the summary receiver operating characteristic curve
HR: Hazard ratio
RR: Risk ratio
CIs: Confidence intervals
