# Supplementary material for: CircRNAs in diagnosis, prognosis, and clinicopathological features of multiple myeloma; a systematic review and meta-analysis
Source: Cancer Cell Int. 2023 Aug 26;23:178. doi: 10.1186/s12935-023-03028-z (PMC10464263; doi:10.1186/s12935-023-03028-z)
Supplement: Supplementary file 1 — Additional file 1: The full text of search strategies for all databases. [file 12935_2023_3028_MOESM1_ESM.docx]

Strategy search formula: **(#1 AND #2) AND (#3 OR #4 OR #5 OR #6 OR #7 OR #8 OR #9 OR #10 OR #11 OR #12 OR #13 OR #14)**

**Search syntax for pubmed**

1

RNA, Circular[mh] OR CircRNAs[tiab] OR Closed Circular RNA[tiab] OR Circular RNA, Closed[tiab] OR RNA, Closed Circular[tiab] OR Circular RNA*[tiab] OR RNAs, Circular[tiab] OR circRNA[tiab] OR Circular Intronic RNA[tiab] OR Intronic RNA, Circular[tiab] OR RNA, Circular Intronic[tiab] OR ciRNA[tiab] OR hsa circ[tiab]

2

Multiple Myeloma[mh] OR Multiple Myelomas[tiab] OR Myelomas, Multiple[tiab] OR Myeloma, Multiple[tiab] OR Myeloma, Plasma-Cell[tiab] OR Myeloma, Plasma Cell[tiab] OR Myelomas, Plasma-Cell[tiab] OR Plasma-Cell Myeloma*[tiab] OR Myelomatosis[tiab] OR Myelomatoses[tiab] OR Plasma Cell Myeloma*[tiab] OR Cell Myeloma, Plasma[tiab] OR Cell Myelomas, Plasma[tiab] OR Myelomas, Plasma Cell[tiab] OR Kahler Disease[tiab] OR Disease, Kahler[tiab] OR Myeloma-Multiple*[tiab]

3

Clinicopathologic*[tiab] OR clinical-pathological characteristics[tiab] OR clinical-pathologic characteristics[tiab]

4

Diagnosis[mh] OR Diagnos*[tiab] OR Diagnoses and Examination*[tiab] OR Examinations and Diagnos*[tiab] OR Postmortem Diagnos*[tiab] OR Diagnoses, Postmortem[tiab] OR Diagnosis, Postmortem[tiab] OR Antemortem Diagnos*[tiab] OR Diagnoses, Antemortem[tiab] OR Diagnosis, Antemortem[tiab]

5

Sensitivity and Specificity[mh] OR Specificity and Sensitivity[tiab] OR Sensitivity[tiab] OR Specificity[tiab]

6

ROC Curve[mh] OR Curve, ROC[tiab] OR Curves, ROC[tiab] OR ROC Curves[tiab] OR Analysis, ROC[tiab] OR Analyses, ROC[tiab] OR ROC Analys*[tiab] OR Receiver Operating Characteristic*[tiab] OR Characteristic, Receiver Operating[tiab] OR Characteristics, Receiver Operating[tiab]

7

Prognosis[mh] OR Prognoses[tiab] OR Prognostic Factor*[tiab] OR Factor, Prognostic[tiab] OR Factors, Prognostic[tiab] OR Progression[tiab]

8

hazard ratio[tiab] OR HR[tiab]

9

overall survival[tiab] OR OS[tiab]

10

Disease-Free Survival[mh] OR Disease Free Survival[tiab] OR Survival, Disease-Free[tiab] OR Survival, Disease Free[tiab] OR DFS[tiab] OR EFS[tiab] OR event-free survival[tiab] OR progression-free survival[tiab] OR PFS[tiab]

11

Area Under Curve[mh] OR Area Under Curves[tiab] OR Curve, Area Under[tiab] OR

Curves, Area Under[tiab] OR Under Curve, Area[tiab] OR Under Curves, Area[tiab] OR AUC[tiab]

12

Therapeutics[mh] OR Therapeutic[tiab] OR Therap*[tiab] OR Treatment*[tiab]

13

Disease Progression[mh] OR Disease Progressions[tiab] OR Progression, Disease[tiab] OR Progressions, Disease[tiab] OR Disease Exacerbation[tiab]

14

Risk Stratification[tiab]

**Search syntax for Scopus**

1

TITLE-ABS-KEY(“RNA, Circular” OR “CircRNAs” OR “Closed Circular RNA” OR “Circular RNA, Closed” OR “RNA, Closed Circular” OR “Circular RNA*” OR “RNAs, Circular” OR “circRNA” OR “Circular Intronic RNA” OR “Intronic RNA, Circular” OR “RNA, Circular Intronic” OR “ciRNA” OR “hsa circ”)

2

TITLE-ABS-KEY(“Multiple Myeloma*” OR “Myelomas, Multiple” OR “Myeloma, Multiple” OR “Myeloma, Plasma-Cell” OR “Myeloma, Plasma Cell” OR “Myelomas, Plasma-Cell” OR “Plasma-Cell Myeloma*” OR “Myelomatosis” OR “Myelomatoses” OR “Plasma Cell Myeloma*” OR “Cell Myeloma, Plasma” OR “Cell Myelomas, Plasma” OR “Myelomas, Plasma Cell” OR “Kahler Disease” OR “Disease, Kahler” OR “Myeloma-Multiple*”)

3

TITLE-ABS-KEY(“Clinicopathologic*” OR “clinical-pathological characteristics” OR “clinical-pathologic characteristics”)

4

TITLE-ABS-KEY(“Diagnos*” OR “Diagnoses and Examination*” OR “Examinations and Diagnos*” OR “Postmortem Diagnos*” OR “Diagnoses, Postmortem” OR “Diagnosis, Postmortem” OR “Antemortem Diagnos*” OR “Diagnoses, Antemortem” OR “Diagnosis, Antemortem”)

5

TITLE-ABS-KEY(“Sensitivity and Specificity” OR “Specificity and Sensitivity” OR “Sensitivity” OR “Specificity”)

6

TITLE-ABS-KEY(“ROC Curve” OR “Curve, ROC” OR “Curves, ROC” OR “ROC Curves” OR “Analysis, ROC” OR “Analyses, ROC” OR “ROC Analys*” OR “Receiver Operating Characteristic*” OR “Characteristic, Receiver Operating” OR “Characteristics, Receiver Operating”)

7

TITLE-ABS-KEY(“Prognos*” OR “Prognostic Factor*” OR “Factor, Prognostic” OR “Factors, Prognostic” OR “Progression”)

8

TITLE-ABS-KEY(“hazard ratio” OR “HR”)

9

TITLE-ABS-KEY(“overall survival” OR “OS”)

10

TITLE-ABS-KEY(“Disease-Free Survival” OR “Disease Free Survival” OR “Survival, Disease-Free” OR “Survival, Disease Free” OR “DFS” OR “EFS” OR “event-free survival” OR “progression-free survival” OR “PFS”)

11

TITLE-ABS-KEY(“Area Under Curve*” OR “Curve, Area Under” OR

“Curves, Area Under” OR “Under Curve, Area” OR “Under Curves, Area” OR “AUC”)

12

TITLE-ABS-KEY(“Therapeutic*” OR “Therap*” OR “Treatment*”)

13

TITLE-ABS-KEY(“Disease Progression*” OR “Progression, Disease” OR “Progressions, Disease” OR “Disease Exacerbation”)

14

TITLE-ABS-KEY(“Risk Stratification”)

**Search syntax for Web of Science**

1

TS=(“RNA, Circular” OR “CircRNAs” OR “Closed Circular RNA” OR “Circular RNA, Closed” OR “RNA, Closed Circular” OR “Circular RNA*” OR “RNAs, Circular” OR “circRNA” OR “Circular Intronic RNA” OR “Intronic RNA, Circular” OR “RNA, Circular Intronic” OR “ciRNA” OR “hsa circ”)

2

TS=(“Multiple Myeloma*” OR “Myelomas, Multiple” OR “Myeloma, Multiple” OR “Myeloma, Plasma-Cell” OR “Myeloma, Plasma Cell” OR “Myelomas, Plasma-Cell” OR “Plasma-Cell Myeloma*” OR “Myelomatosis” OR “Myelomatoses” OR “Plasma Cell Myeloma*” OR “Cell Myeloma, Plasma” OR “Cell Myelomas, Plasma” OR “Myelomas, Plasma Cell” OR “Kahler Disease” OR “Disease, Kahler” OR “Myeloma-Multiple*”)

3

TS=(“Clinicopathologic*” OR “clinical-pathological characteristics” OR “clinical-pathologic characteristics”)

4

TS=(“Diagnos*” OR “Diagnoses and Examination*” OR “Examinations and Diagnos*” OR “Postmortem Diagnos*” OR “Diagnoses, Postmortem” OR “Diagnosis, Postmortem” OR “Antemortem Diagnos*” OR “Diagnoses, Antemortem” OR “Diagnosis, Antemortem”)

5

TS=(“Sensitivity and Specificity” OR “Specificity and Sensitivity” OR “Sensitivity” OR “Specificity”)

6

TS=(“ROC Curve” OR “Curve, ROC” OR “Curves, ROC” OR “ROC Curves” OR “Analysis, ROC” OR “Analyses, ROC” OR “ROC Analys*” OR “Receiver Operating Characteristic*” OR “Characteristic, Receiver Operating” OR “Characteristics, Receiver Operating”)

7

TS=(“Prognos*” OR “Prognostic Factor*” OR “Factor, Prognostic” OR “Factors, Prognostic” OR “Progression”)

8

TS=(“hazard ratio” OR “HR”)

9

TS=(“overall survival” OR “OS”)

10

TS=(“Disease-Free Survival” OR “Disease Free Survival” OR “Survival, Disease-Free” OR “Survival, Disease Free” OR “DFS” OR “EFS” OR “event-free survival” OR “progression-free survival” OR “PFS”)

11

TS=(“Area Under Curve*” OR “Curve, Area Under” OR

“Curves, Area Under” OR “Under Curve, Area” OR “Under Curves, Area” OR “AUC”)

12

TS=(“Therapeutic*” OR “Therap*” OR “Treatment*”)

13

TS=(“Disease Progression*” OR “Progression, Disease” OR “Progressions, Disease” OR “Disease Exacerbation”)

14

TS=(“Risk Stratification”)

**Search syntax for Poquest**

1

TI,AB,SU(“RNA, Circular” OR “CircRNAs” OR “Closed Circular RNA” OR “Circular RNA, Closed” OR “RNA, Closed Circular” OR “Circular RNA*” OR “RNAs, Circular” OR “circRNA” OR “Circular Intronic RNA” OR “Intronic RNA, Circular” OR “RNA, Circular Intronic” OR “ciRNA” OR “hsa circ”)

2

TI,AB,SU(“Multiple Myeloma*” OR “Myelomas, Multiple” OR “Myeloma, Multiple” OR “Myeloma, Plasma-Cell” OR “Myeloma, Plasma Cell” OR “Myelomas, Plasma-Cell” OR “Plasma-Cell Myeloma*” OR “Myelomatosis” OR “Myelomatoses” OR “Plasma Cell Myeloma*” OR “Cell Myeloma, Plasma” OR “Cell Myelomas, Plasma” OR “Myelomas, Plasma Cell” OR “Kahler Disease” OR “Disease, Kahler” OR “Myeloma-Multiple*”)

3

TI,AB,SU(“Clinicopathologic*” OR “clinical-pathological characteristics” OR “clinical-pathologic characteristics”)

4

TI,AB,SU(“Diagnos*” OR “Diagnoses and Examination*” OR “Examinations and Diagnos*” OR “Postmortem Diagnos*” OR “Diagnoses, Postmortem” OR “Diagnosis, Postmortem” OR “Antemortem Diagnos*” OR “Diagnoses, Antemortem” OR “Diagnosis, Antemortem”)

5

TI,AB,SU(“Sensitivity and Specificity” OR “Specificity and Sensitivity” OR “Sensitivity” OR “Specificity”)

6

TI,AB,SU(“ROC Curve” OR “Curve, ROC” OR “Curves, ROC” OR “ROC Curves” OR “Analysis, ROC” OR “Analyses, ROC” OR “ROC Analys*” OR “Receiver Operating Characteristic*” OR “Characteristic, Receiver Operating” OR “Characteristics, Receiver Operating”)

7

TI,AB,SU(“Prognos*” OR “Prognostic Factor*” OR “Factor, Prognostic” OR “Factors, Prognostic” OR “Progression”)

8

TI,AB,SU(“hazard ratio” OR “HR”)

9

TI,AB,SU(“overall survival” OR “OS”)

10

TI,AB,SU(“Disease-Free Survival” OR “Disease Free Survival” OR “Survival, Disease-Free” OR “Survival, Disease Free” OR “DFS” OR “EFS” OR “event-free survival” OR “progression-free survival” OR “PFS”)

11

TI,AB,SU(“Area Under Curve*” OR “Curve, Area Under” OR

“Curves, Area Under” OR “Under Curve, Area” OR “Under Curves, Area” OR “AUC”)

12

TI,AB,SU(“Therapeutic*” OR “Therap*” OR “Treatment*”)

13

TI,AB,SU(“Disease Progression*” OR “Progression, Disease” OR “Progressions, Disease” OR “Disease Exacerbation”)

14

TI,AB,SU(“Risk Stratification”)
