# Supplementary material for: CircRNAs in diagnosis, prognosis, and clinicopathological features of multiple myeloma; a systematic review and meta-analysis
Source: Cancer Cell Int. 2023 Aug 26;23:178. doi: 10.1186/s12935-023-03028-z (PMC10464263; doi:10.1186/s12935-023-03028-z)
Supplement: Supplementary file 2 — Additional file 2: Figure S1. Quality assessment by the QUADAS II. Each bias risk item for each included study (A), each bias risk item is presented as a percentage for all included studies (B). Table S1. Supplemental Content, which illustrates study quality assessed via the Newcastle-Ottawa Scale checklist. [file 12935_2023_3028_MOESM2_ESM.docx]

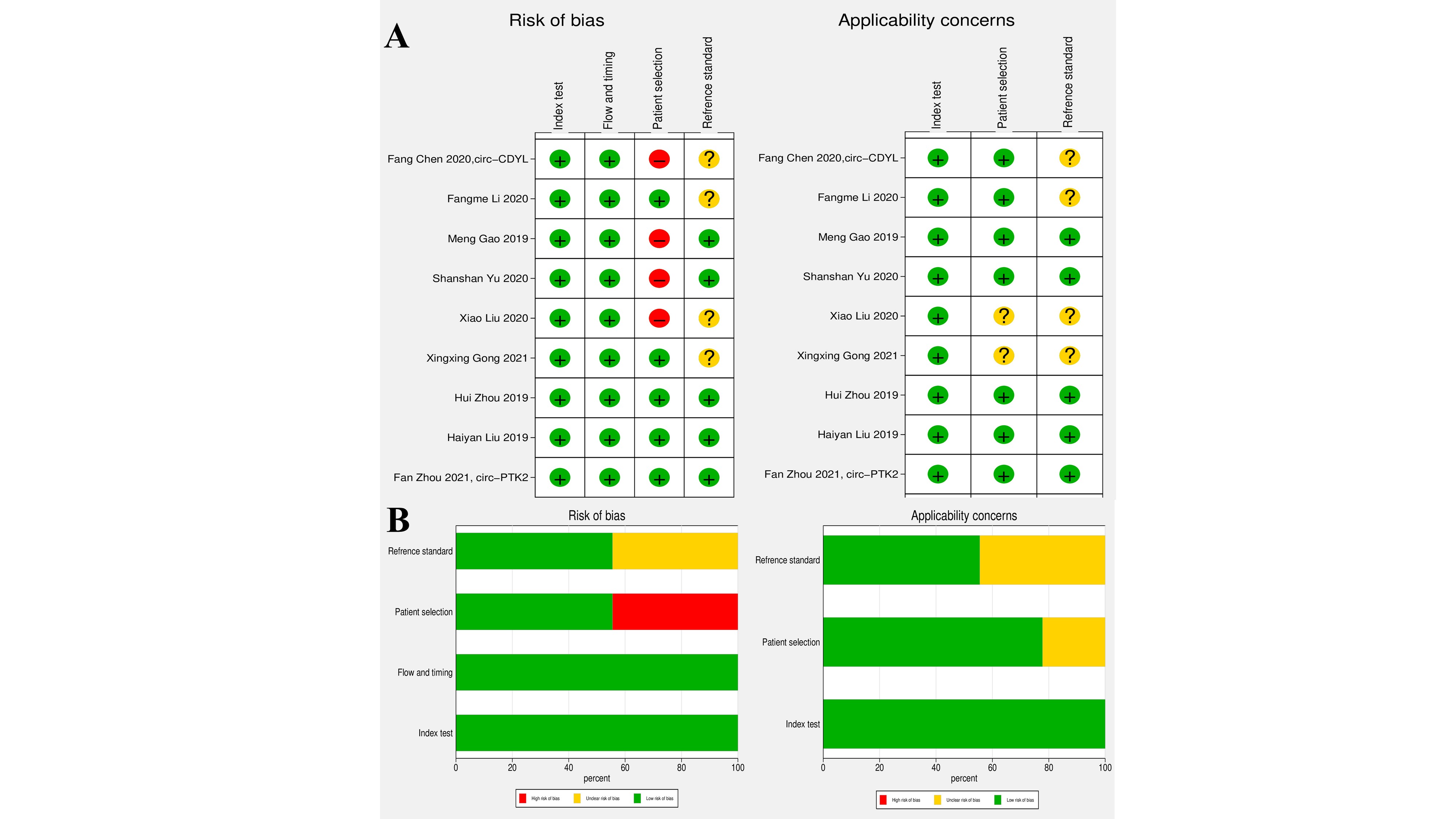


**Figure S1.** **Quality assessment by the QUADAS II**. Each bias risk item for each included study (A), each bias risk item is presented as a percentage for all included studies (B).

**Table S1** Supplemental Content, which illustrates study quality assessed via the Newcastle-Ottawa Scale checklist

| Study | Selection | Comparability | Outcome | Total score |
| --- | --- | --- | --- | --- |
| Fang Chen 2020  circ-CDYL | **☆☆☆☆** | **☆** | **☆☆** | 7 |
| Haiyan Liu 2019 | **☆☆☆☆** | **☆☆** | **☆☆** | 8 |
| Hui Zhou 2019 | **☆☆☆☆** | **☆☆** | **☆☆** | 8 |
| Shanshan Yu 2020 | **☆☆☆☆** | **☆** | **☆** | 6 |
| Xiao Liu 2020 | **☆☆☆☆** | **☆** | **☆☆** | 7 |
| Xingxing Gong 2021 | **☆☆☆☆** | **☆☆** | **☆** | 7 |
| Fang Chen2020  Circ0069767 | **☆☆☆☆** | **☆** | **☆☆** | 7 |
| Hongyan Ma 2022 | **☆☆☆☆** | **☆** | **☆☆** | 7 |
| Lin Liu 2021 | **☆☆☆☆** | **☆☆** | **☆☆** | 8 |
| Yanwei Luo 2020 | **☆☆☆☆** | **☆☆** | **☆☆** | 8 |
| Yashu Feng 2019 | **☆☆☆☆** | **☆** | **☆** | 6 |
| Yongsheng Xiang2021 | **☆☆☆☆** | **☆☆** | **☆** | 7 |
| Fan Zhou 2021 | **☆☆☆☆** | **☆** | **☆** | 6 |
| Fang Liu 2020 | **☆☆☆☆** | **☆☆** | **☆☆** | 8 |
| Wei Fang 2021 | **☆☆☆☆** | **☆☆** | **☆☆** | 8 |
| Manya Yu 2022 | **☆☆☆☆** | **☆** | **☆** | 6 |
| Runjie Sun 2021 | **☆☆☆☆** | **☆☆** | **☆** | 7 |
| Xiaoya Li 2021 | **☆☆☆** | **☆** | **☆☆** | 7 |
| Yan Wang 2021 | **☆☆☆** | **☆** | **☆☆** | 7 |
| Xiaozhu Tang 2022  circHNRNPU | **☆☆☆☆** | **☆** | **☆** | 6 |
| Yu-Hui Zhu 2021 | **☆☆☆** | **☆** | **☆☆** | 7 |
| Yan Li 2022 | **☆☆☆☆** | **☆☆** | **☆☆** | 8 |
| Fangmei Li 2022 | **☆** | **☆☆** | **☆☆** | 5 |
| Xiaozhu Tang 2021  circBUB1B | **☆☆☆☆** | **☆** | **☆** | 6 |
| Meng Gao 2019 | **☆☆** | **☆☆** | **☆☆** | 8 |
| Jianhua Liu 2020 | **☆☆☆☆** | **☆** | **☆☆** | 7 |
| Lianguo Xue 2021 | **☆☆☆☆** | **☆** | **☆☆** | 7 |
