# Supplementary material for: CircRNAs in diagnosis, prognosis, and clinicopathological features of multiple myeloma; a systematic review and meta-analysis
Source: Cancer Cell Int. 2023 Aug 26;23:178. doi: 10.1186/s12935-023-03028-z (PMC10464263; doi:10.1186/s12935-023-03028-z)
Supplement: Supplementary file 3 — Additional file 3: Figure S1. Likelihood ratio scattergram (A), Relationship between pre and post-test probability based on the likelihood of a positive (above digonal line) or negative (below diagonal line) test (B), Fagan’s nomogram to describe the effect of circRNAs on the diagnosis of MM (C). Figure S2. Forest plots of Subgroup analysis based on DOR. Subgroup analysis based on type of circRNAs (A), Subgroup analysis based on quadas score (B). [file 12935_2023_3028_MOESM3_ESM.docx]

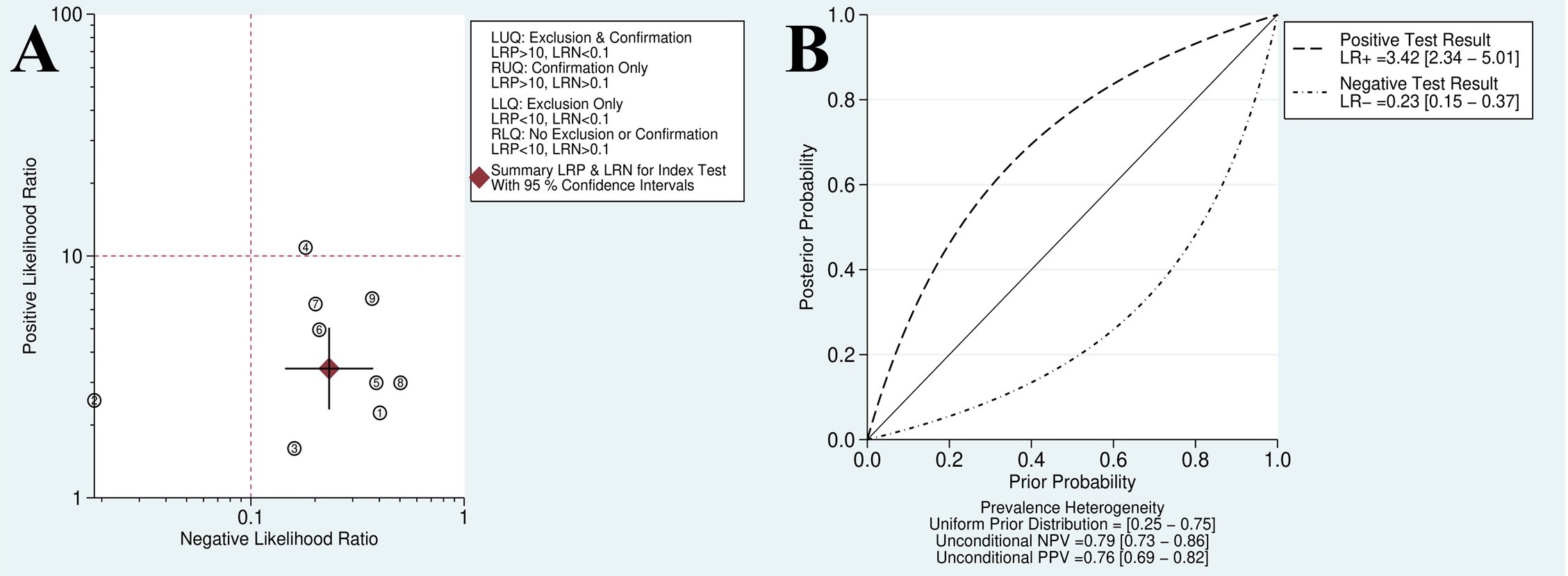


**C**

**Figure 1.** Likelihood ratio scattergram (A), Relationship between pre and post-test probability based on the likelihood of a positive (above digonal line) or negative (below diagonal line) test (B), Fagan’s nomogram to describe the effect of circRNAs on the diagnosis of MM (C).


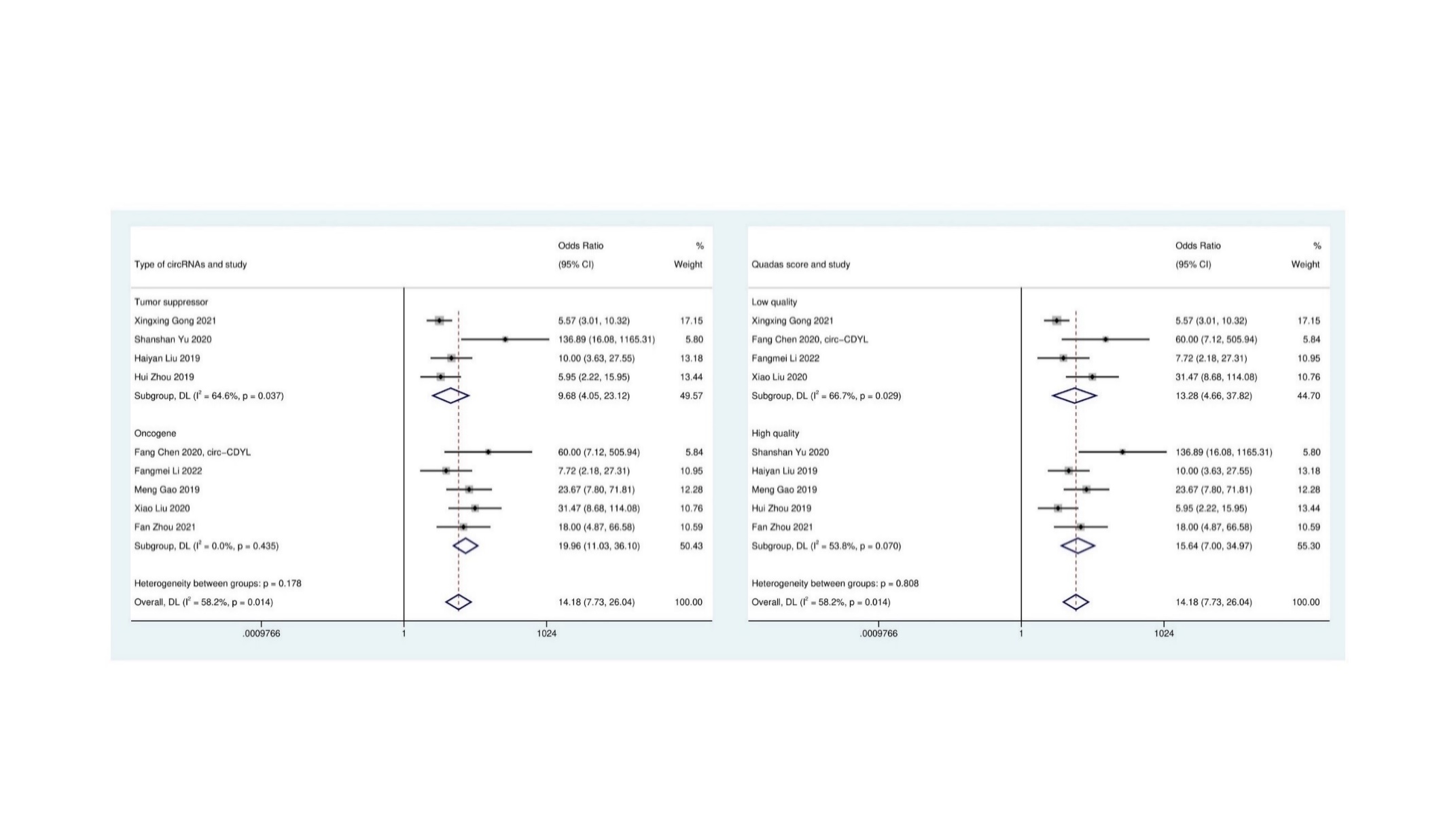


**Figure 2.** **Forest plots of Subgroup analysis based on DOR.** Subgroup analysis based on type of circRNAs (A), Subgroup analysis based on quadas score (B).

**A**

**B**
