# Supplementary material for: CircRNAs in diagnosis, prognosis, and clinicopathological features of multiple myeloma; a systematic review and meta-analysis
Source: Cancer Cell Int. 2023 Aug 26;23:178. doi: 10.1186/s12935-023-03028-z (PMC10464263; doi:10.1186/s12935-023-03028-z)

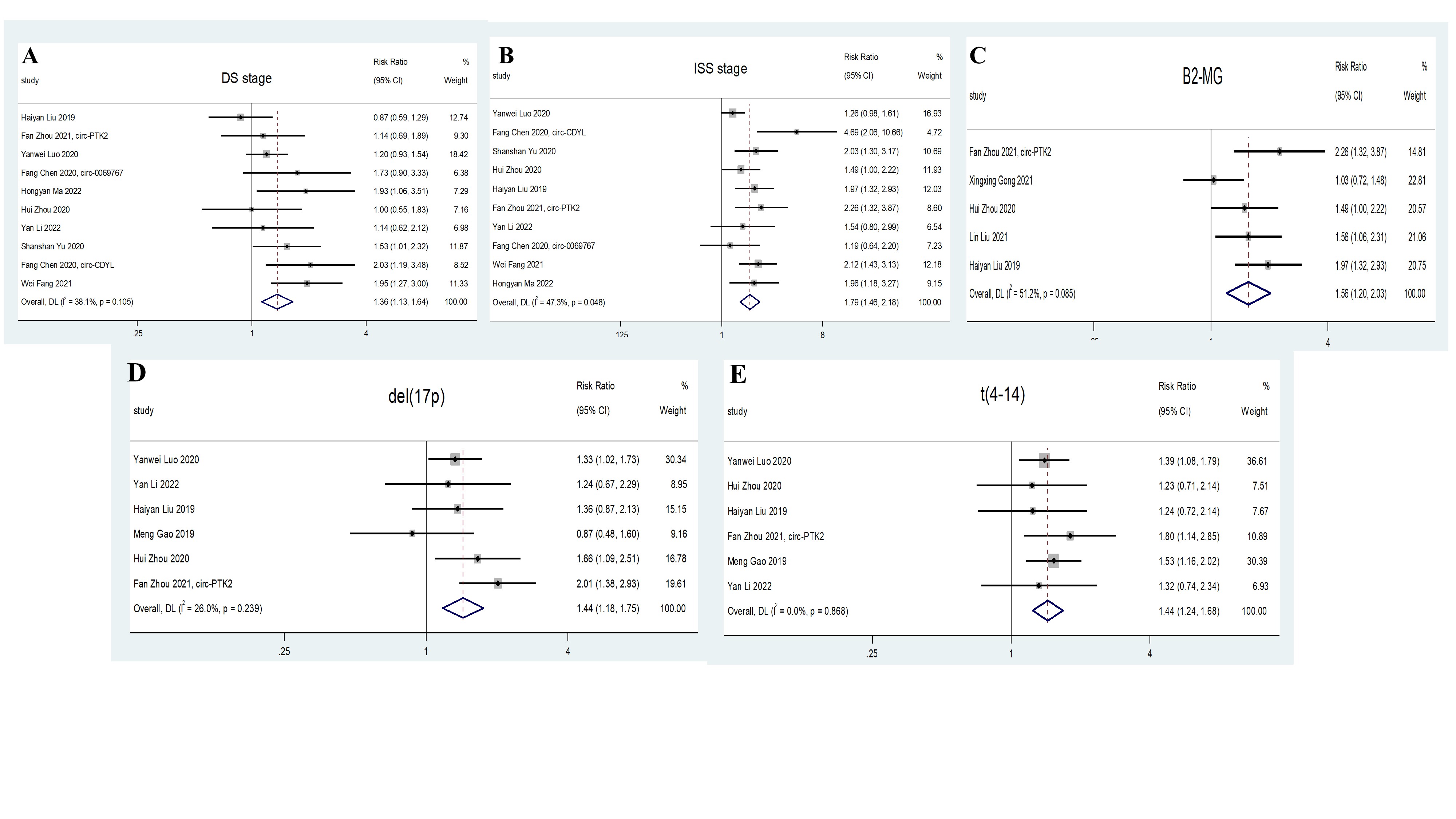


**Figure 1.** Forest plots of DS stage (A), ISS stage (B), B2-MG (C), del(17p) (D) and t(4;14) (E) in the clinicopathological features association analysis with circRNAs in MM patients.

**Figure 2.** Forest plots of other clinicopathological parameters


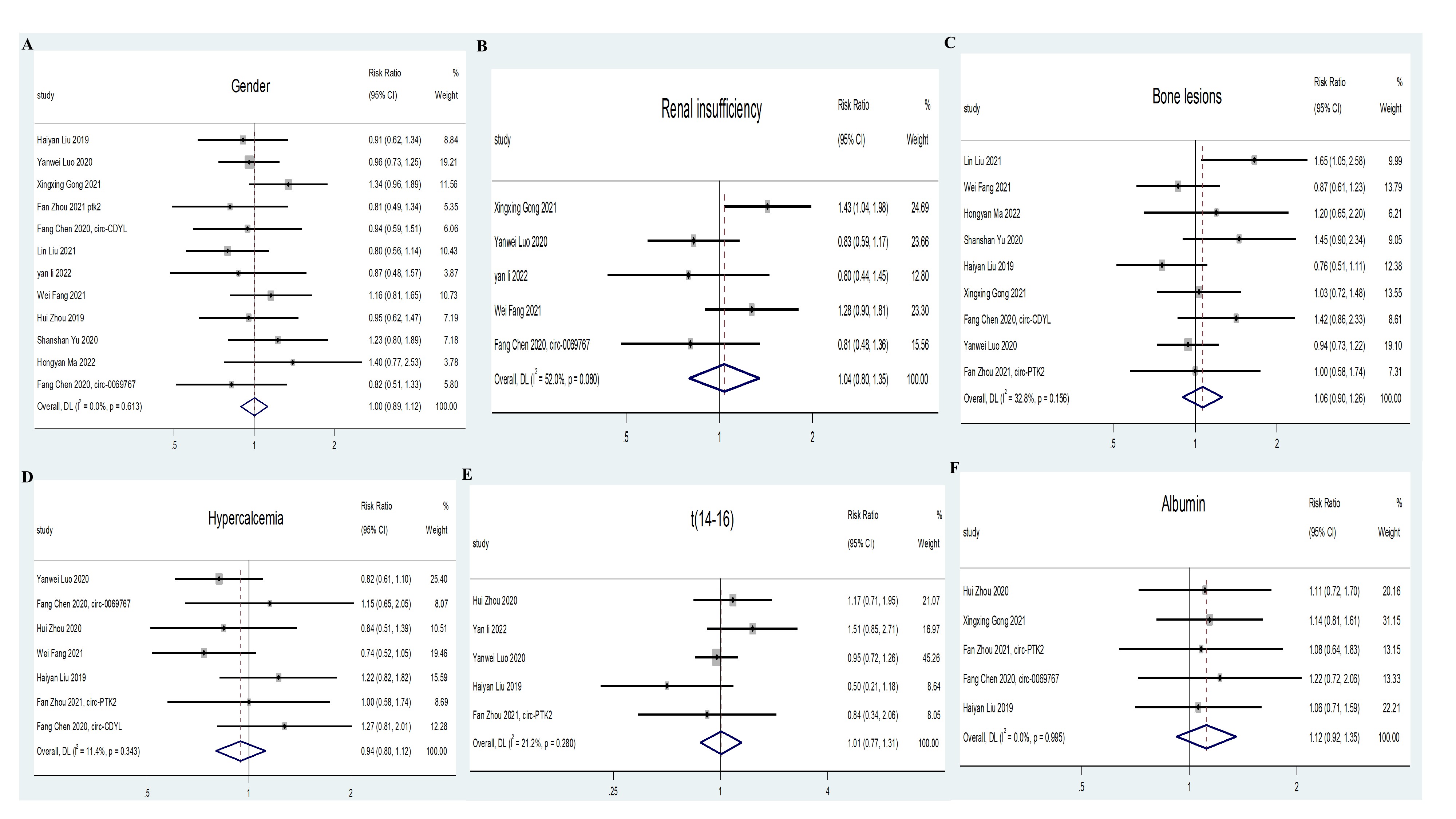

Supplement: Supplementary file 4 — Additional file 4: Figure S1. Forest plots of DS stage (A), ISS stage (B), B2-MG (C), del(17p) (D) and t(4;14) (E) in the clinicopathological features association analysis with circRNAs in MM patients. Figure S2 Forest plots of other clinicopathological parameters. [file 12935_2023_3028_MOESM4_ESM.docx]
