# Supplementary material for: CircRNAs in diagnosis, prognosis, and clinicopathological features of multiple myeloma; a systematic review and meta-analysis
Source: Cancer Cell Int. 2023 Aug 26;23:178. doi: 10.1186/s12935-023-03028-z (PMC10464263; doi:10.1186/s12935-023-03028-z)
Supplement: Supplementary file 5 — Additional file 5: Figure S1. Publication bias evaluation for prognostic studies. Egger’s test (A) and Trim and fill (B) method for oncogene circRNAs. Egger’s test (C) and Trim and fill (D) method for tumor suppressor circRNAs. Figure S2. Sensitivity analysis for oncogene (A) and tumor suppressor (B) circRNAs.Figure S3. Sensitivity analysis (A) and Deeks’ funnel plot (B) for diagnostic studies. [file 12935_2023_3028_MOESM5_ESM.docx]

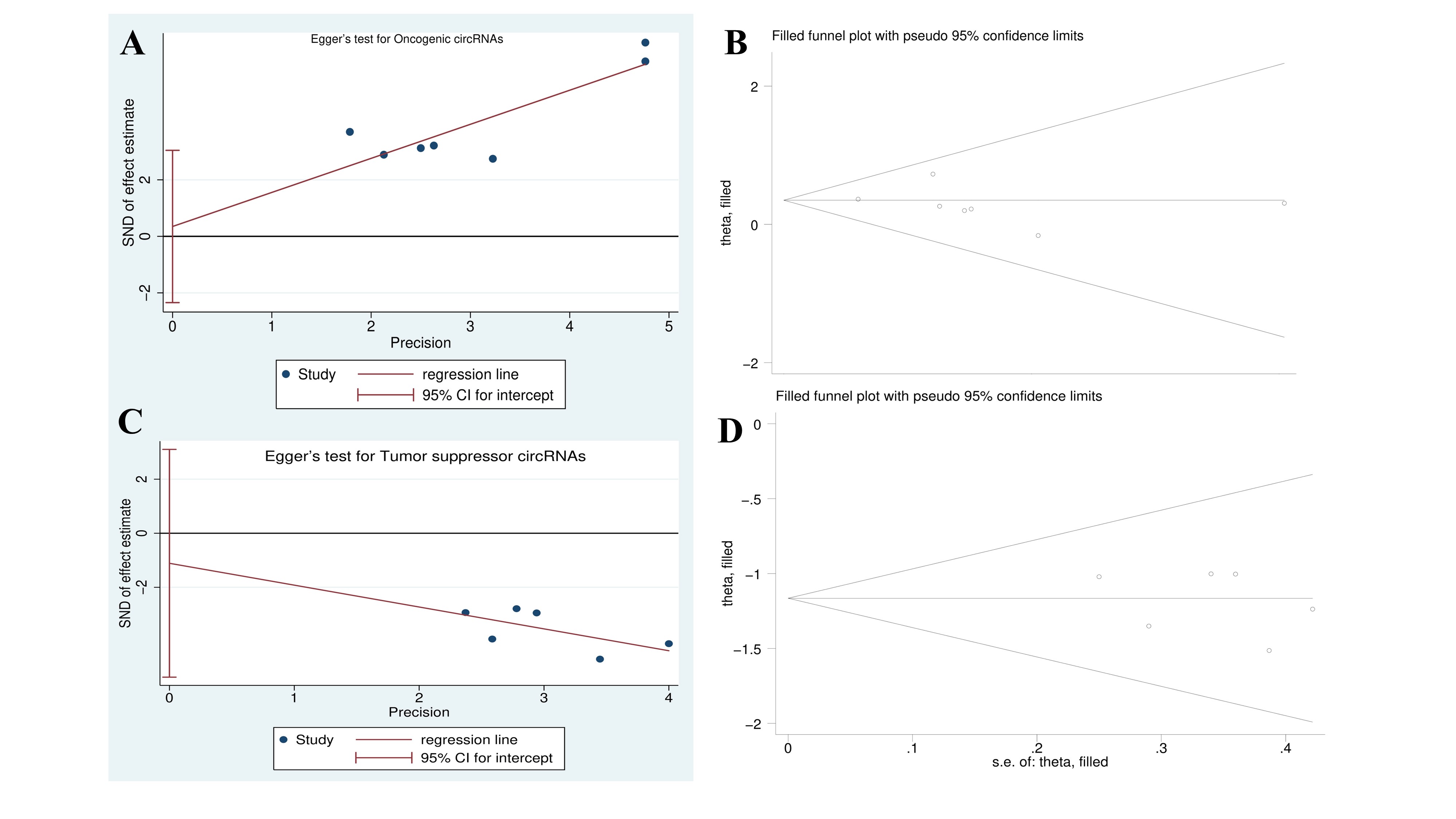


**Figure 1.** Publication bias evaluation for prognostic studies. Egger’s test (A) and Trim and fill (B) method for oncogene circRNAs. Egger’s test (C) and Trim and fill (D) method for tumor suppressor circRNAs.


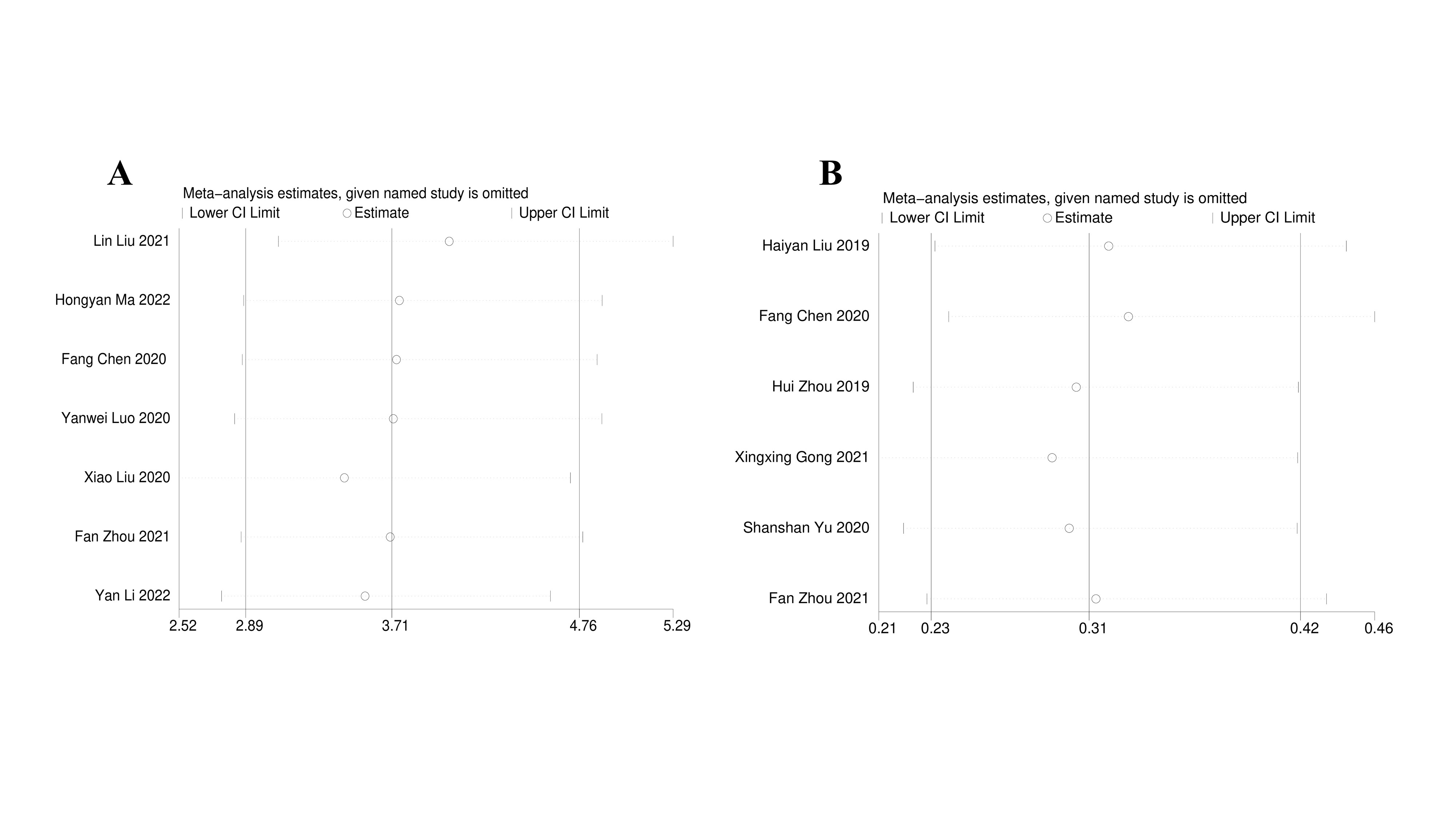


**Figure 2.** Sensitivity analysis for oncogene (A) and tumor suppressor (B) circRNAs.


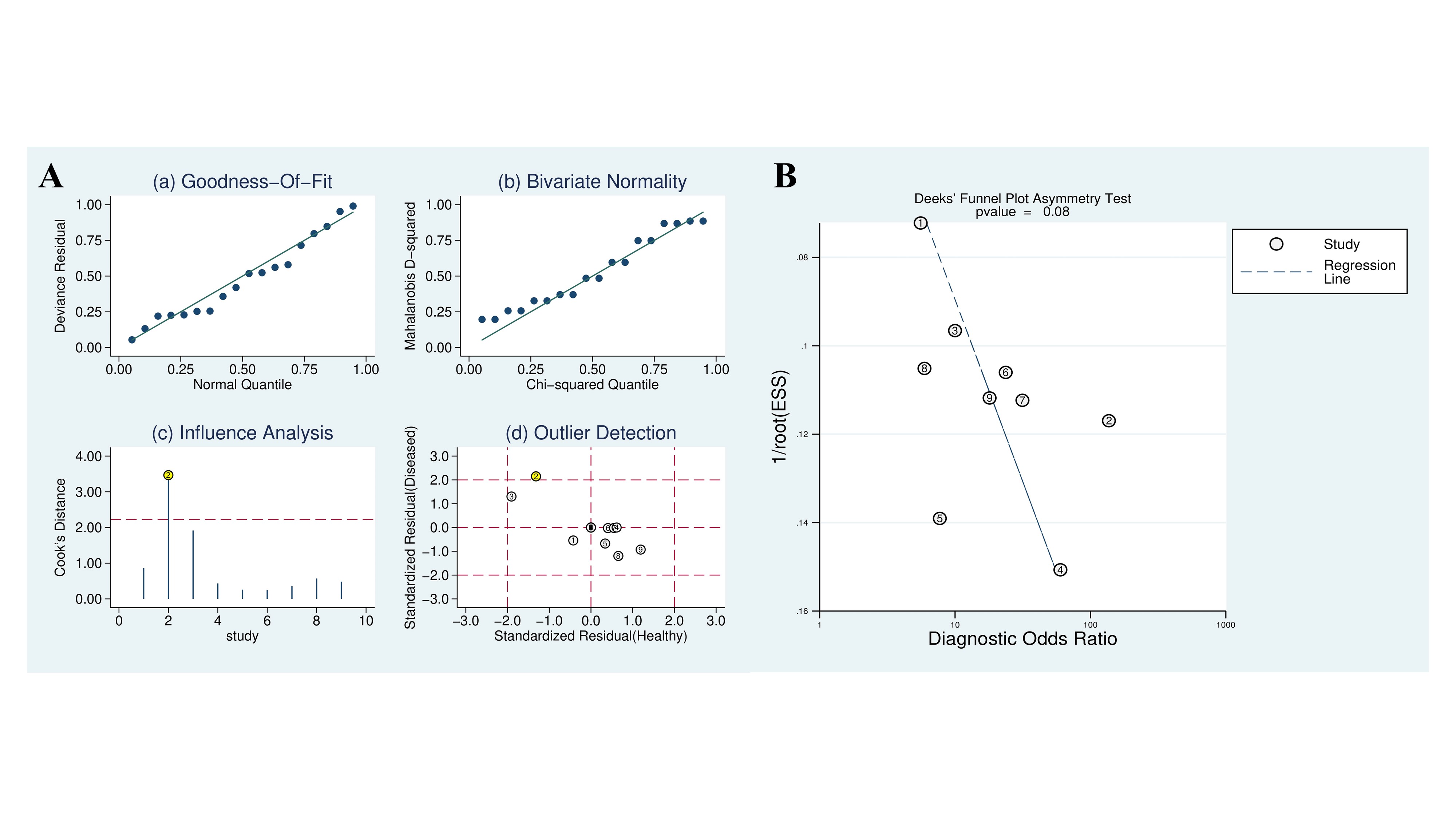


**Figure 3.** Sensitivity analysis (A) and Deeks’ funnel plot (B) for diagnostic studies.
